# Incidental deep venous thrombosis diagnosed on lower extremity computed tomography is a rare but clinically impactful finding

**DOI:** 10.1016/j.jvsv.2024.101858

**Published:** 2024-03-05

**Authors:** Peter A.L. Barros, Daniel J. Castro, Roger E. Goldman, Mimmie Kwong

**Affiliations:** a University of California Davis School of Medicine, Sacramento, CA; b Division of Interventional Radiology, Department of Radiology, University of California Davis School of Medicine, Sacramento, CA; c Division of Vascular Surgery, Department of Surgery, University of California Davis School of Medicine, Sacramento, CA

**Keywords:** Deep vein thrombosis, Venous thromboembolism, Computed tomography, Ultrasound, Incidental findings

## Abstract

**Background:**

In the setting of a known thrombotic event, computed tomography (CT) studies provide reasonable sensitivity for the diagnosis of deep venous thrombosis (DVT). However, the incidence and accuracy of a DVT diagnosis on CT studies not targeted for the detection of DVT are not well described. In addition, the clinical impact of DVTs incidentally identified on CT is unknown.

**Methods:**

In this single-institution retrospective study, we queried all contrasted CT studies of the lower extremities performed over a 10-year period. Regular expressions applied to the radiology reports associated with the CT studies identified studies with positive findings associated with DVT. These selected reports were then manually reviewed to confirm the presence of a DVT. Patient demographics and relevant medical and surgical history were obtained through a chart review. Follow-up information was obtained for 1 year after the incident CT and included treatment course, additional imaging, and adverse events. An incidental DVT was one identified in a patient in whom the DVT was not noted in a prior study and for whom the study indication did not include concern for DVT or pulmonary embolism.

**Results:**

Of 16,637 lower extremity contrasted CT studies queried, 37 study reports identified a DVT. However, only 13 patients had a finding of an incidental DVT (10-year incidence of 0.08%). Among these 13 patients, 11 underwent additional imaging, including 9 who had a subsequent venous duplex and 2 who had subsequent CT studies. Among those with a subsequent duplex, DVT was not identified in eight cases, whereas in one case, DVT was confirmed. Among those with subsequent CT studies, DVT was not identified in one case and was confirmed in one case. Of the 13 patients with incidental DVTs, 3 were initiated on anticoagulation based on their initial CT findings alone. Among these, two did not experience any complications from their DVT or anticoagulation regimen. One did experience major bleeding complications, requiring additional procedures.

**Conclusions:**

Incidental DVTs are a rare finding in lower extremity CT studies, noted to occur in only 0.08% of studies. Most patients with incidental DVTs receive additional imaging, with negative findings in 80% of cases. This study identified that 23% of patients were initiated on anticoagulation due to the CT findings, with a 33% rate of significant complications. Currently, a CT venogram is not recommended as a first-line modality for the diagnosis of DVT. However, there is no guidance regarding the need for repeat imaging in patients with incidentally diagnosed lower extremity DVTs identified on CT. Additional study is needed to provide evidence for guideline development.

**Article Highlights**

• **Type of Research:** Single-institution retrospective study
• **Key Findings:** Among 16,637 contrasted computed tomography (CT) studies of the lower extremity, 37 identified a deep venous thrombosis (DVT). However, only 13 patients had truly incidental DVTs (10-year incidence of 0.08%). Of these 13 patients, 3 were initiated on anticoagulation based on CT findings; 2 of these patients had no complications, whereas 1 experienced major bleeding complications, requiring additional procedures.
• **Take Home Message:** Incidental diagnoses of DVT by contrasted lower extremity CT are rare but may negatively affect patient outcomes. Confirmatory studies may be beneficial after incidental DVT diagnosis, but additional investigation with larger multicenter studies is needed to provide evidence for guideline development.

Venous thromboembolism (VTE), the condition comprising deep venous thrombosis (DVT) and pulmonary embolism (PE), remains a relatively common and life-threatening medical condition.^1^ The United States Centers for Disease Control estimates that 900,000 people per year are diagnosed with a PE and/or DVT with VTE causing 60 to 100,000 deaths annually. The diagnosis of either DVT or PE often warrants reflex workup for the other, as approximately 50% of patients diagnosed with DVT are then found to have PE and approximately 70% of patients diagnosed with PE are then found to have DVT.^2^ Although most of the mortality directly associated with VTE is due to PE, DVT is not a benign disease. Six percent of patients diagnosed with DVT die within the first month of diagnosis, signifying both the risk for progression to destabilizing PE and the tenuous health of many patients diagnosed with DVT.^3^ Patients with DVT can also have significant morbidity, with one-third of patients developing post-thrombotic syndrome.^4^ DVT diagnosis and treatment are also associated with significant health care costs ranging between $5 and $8 billion per year or approximately $20,000 per patient in the United States.^5^ Central (iliofemoral) DVT remains especially concerning due to its propensity for causing clinically significant PE and post-thrombotic syndrome.^6^^,^^7^ However, even in peripheral (calf) DVTs, there is an approximately 20% risk for proximal propagation and a 50% likelihood of concurrent PE diagnosis.^2^ Therefore, the initiation of appropriate antithrombotic therapy in patients diagnosed with DVT is paramount.

Diagnostic criteria and assessment of DVT require objective measurements such as D-dimer or ultrasound (US) imaging as the physical examination alone does not provide sufficient sensitivity and specificity.^6^^,^^7^ Historically, conventional venography was the gold standard for diagnosis.^6^, ^7^, ^8^ However, venography is an invasive procedure with associated risks. As a result, this diagnostic modality has been supplanted by US imaging, which has been noted to have reasonable sensitivity and specificity for the diagnosis of a suspected DVT. US imaging is noted to have sensitivities of 89% to 96% and specificities of 94% to 99% for the diagnosis of DVT.^9^ Current CHEST guidelines do not recommend the routine use of CT (computerized tomography) for the diagnosis of DVT, though studies suggest that a CT venogram has sensitivities of 89% to 100% and specificities of 94% to 100% as a diagnostic modality for DVT.^10^

Although both CT and US imaging are used in the evaluation for DVT, they each have specific limitations. For US imaging, imaging quality and completeness are operator dependent and can be affected by patient factors such as body habitus, discomfort, and compliance. CT provides excellent axial resolution, but adequate contrast opacification for evaluation of luminal patency faces multiple technical limitations, including patient (eg, age, body habitus, and cardiac output), contrast (eg, injection rate and iodine mass), and study (eg, scan delay and scan duration) factors, of which only some are controllable.^11^

Although the use of CT imaging has proven to be reasonably effective in diagnosing patients with suspected VTE in the right clinical scenario, there are limited data on the incidence and diagnostic accuracy of DVTs identified incidentally on CT studies of the lower extremity that were not specifically protocoled to assess the venous circulation.^12^ In addition, it is unknown how these incidentally diagnosed DVTs affect patient care and outcomes.

## Methods

This retrospective review of institutional data was approved by the University of California, Davis Institutional Review Board. An institutional imaging database was queried for all patients who underwent contrasted CT of the lower extremity between the years 2010 and 2020. CT lower extremity studies with at least one of the keywords “DVT,” “thrombosis,” or “clot” in the CT report and no US lower extremity study within the prior 30 days were identified using Structured Query Language (SQL) queries with regular expressions. CT reports were reviewed to identify studies where DVT was mentioned in the main body of the report or within the findings/impression documented by a radiologist. For each patient with a CT report that identified a DVT, the electronic medical record was reviewed to identify patient demographics (age, gender, race, and insurance status) and comorbidities (atrial fibrillation, congestive heart failure, end-stage renal disease, and chronic kidney disease). Conditions and medications relevant to thrombosis risk were also assessed (history of DVT, PE, superficial vein thrombosis, varicose veins, lower extremity vein treatment, inferior vena cava [IVC] filter placement, hypercoagulable disorder, hematologic or solid organ malignancy, ipsilateral extremity trauma or surgery, and use of anticoagulants and/or antiplatelets).

CT reports were reviewed to confirm the location of the DVT (IVC, common iliac, internal iliac, external iliac, common femoral, femoral, deep femoral, popliteal, or tibial veins). In addition, the type of CT ordered (CT with contrast, CT with/without contrast, or CT angiogram), the indication for the study, and the details of the ordering provider (including type, clinical specialty, and location [emergency department, outpatient, and inpatient]) were assessed. The clinical course over the subsequent 365 days from the initial CT study was reviewed for VTE-related management, treatment, and outcomes. This included a review of the timing and findings of any subsequent imaging of the lower extremity veins by US imaging and CT, type and duration of any anticoagulation, and complications. Complications were defined as progression to more proximal DVT or PE, minor bleeding (clinically documented bleeding—such as a hematoma—with no change clinical course or intervention required), and major bleeding (life-threatening or bleeding requiring any type of intervention, including additional procedures, transfusion, and transfer to the intensive care unit [ICU]) events, stroke/transient ischemic attack, and death.

An incidentally found DVT was defined as a DVT that was (1) identified on a CT scan for which the study indication did not mention DVT or PE and (2) done for a patient with no known DVT in that location based on a chart review, including reports from prior imaging.

## Results

A total of 16,637 contrasted lower extremity CT studies were performed at the University of California, Davis, between 2010 and 2020. A total of 125 radiology reports had keyword matches. Of these, 37 positively identified a DVT, corresponding to a 10-year incidence of 0.22% for any DVTs identified on CT of the lower extremity.

Among the 37 patients with DVTs, the mean age was found to be 50 ± 15.5 years. There was a slight male preponderance (57% males vs 43% females). The majority of patients were White (70%). Most patients (81.08%) had some form of insurance, with 15 (41%) having private insurance, 7 (19%) having Medicare, and 6 (16%) having Medicaid/MediCal. Over half (51%) of the patients had a prior history of DVT, with most (17) involving the ipsilateral limb. Furthermore, six (16%) patients had a history of IVC filter placement, seven (19%) had a history of PE, two (5%) had a history of hypercoagulable disorder, and one (3%) had a history of superficial vein thrombosis. A large portion of the patients had an active malignancy, with seven (19%) having a solid organ malignancy. Five (14%) patients had a history of ipsilateral orthopedic trauma and seven (19%) had a history of ipsilateral orthopedic surgery. Nineteen percent of patients were on aspirin at the time of the CT study, and 49% (18) were on therapeutic anticoagulation. The most common anticoagulant used was warfarin (eight patients) (Table I).

**Table I:** Distribution of patient factors among patients with DVT identified on CT scan

Patient factors	Value (N = 37)
Age, years, mean ± SD	50 ± 15.5
Gender, No. (%)	
Male	21 (56.8)
Female	16 (43.2)
Race, No. (%)	
White	26 (70.3)
Black	5 (13.5)
Hispanic/Latino	4 (10.8)
Asian/Pacific Islander	2 (5.4)
Insurance, No. (%)	
Medicare	7 (18.9)
Medicaid/MediCal	6 (16.2)
Private insurance	15 (40.5)
VA/TRICARE	0
Other	2 (5.4)
Past venous history, No. (%)	
Pulmonary embolism	7 (18.9)
Deep venous thrombosis (DVT)	19 (51.4)
Ipsilateral DVT	17 (45.9)
Superficial vein thrombosis	1 (2.7)
Ipsilateral superficial vein thrombosis	1 (2.7)
Varicose veins	0
Prior vein treatment	0
IVC filter placement	6 (16.2)
Past medical and surgical history	
Solid organ malignancy	7 (19.4)
Active solid organ malignancy	7 (19.4)
Hematologic malignancy	2 (5.4)
Active hematologic malignancy	2 (5.4)
Atrial fibrillation	0
Congestive heart failure	3 (8.1)
Chronic kidney disease	1 (2.7)
End-stage renal disease	0
Ipsilateral orthopedic trauma	5 (13.5)
Ipsilateral orthopedic surgery	7 (18.9)
Hypercoagulable disorder	2 (5.7)
Medications, No. (%)	
Aspirin	7 (20.6)
Clopidogrel	0
Other antiplatelet	0
Anticoagulation	18 (48.6)
Warfarin	8 (21.6)
Apixaban	4 (10.8)
Rivaroxiban	4 (10.8)
Dabigatran	0
Enoxaparin	2 (5.4)
Fondaparinux	1 (2.7)
Other	1 (2.7)
Chronic/known DVT, No. (%)	22 (59.5)

*IVC*, Inferior vena cava; *SD*, standard deviation; *VA*, Veterans Affairs.

DVTs were noted most commonly in the common femoral vein (43%), followed by the external iliac vein (38%), femoral vein (38%), and the popliteal vein (32%) (Table II).

**Table II:** Distribution of deep venous thrombosis (*DVT*) locations

DVT locations	Value, No. (%)
Inferior vena cava	8 (21.6)
Common iliac vein	7 (18.9)
Internal iliac vein	3 (8.1)
External iliac vein	14 (37.8)
Common femoral vein	16 (43.2)
Deep (profunda) femoral vein	6 (16.2)
(Superficial) femoral vein	14 (37.8)
Popliteal vein	12 (32.4)
Tibial vein	2 (5.4)

Of the 37 patients, 13 had DVTs that were incidentally found, corresponding to a 10-year incidence rate of 0.08%. Four of the 13 patients were noted to have an active malignancy, one had a history of prior ipsilateral DVT, and one was on therapeutic anticoagulation at the time of the CT scan. The indications documented for these CT studies included infection (62%), pain/swelling (31%), and bleeding/hematomas (8%). The most common site for thrombosis was the femoral vein (46%), followed by the common femoral vein (31%), external iliac vein (23%), and popliteal vein (23%) (Table III). Half (6 of 13) of the patients had nonocclusive thrombus, whereas the remaining patients had thrombus that was occlusive distally but partially occlusive proximally. Examples of the images identifying the incidental DVT are noted in the Fig.

**Table III:** Summary of patient and imaging factors, additional workup, and treatments among patients with incidental deep venous thromboses (*DVTs*) found on computed tomography (*CT*) scan

Patient	Relevant history	Indication	DVT location	Subsequent imaging	Timing after CT	Results	Anticoagulation
23	–	Infection	External iliac and common femoral veins	Ultrasound	3 days	Negative	None
40	Prior ipsilateral DVT, on anticoagulation	Pain	Deep femoral vein	CT	3 days	Positive	Yes
53	–	Infection	External iliac and common femoral veins	Ultrasound	Same day	Negative	None
55	Active malignancy	Pain	Left popliteal vein	Ultrasound	2 days	Negative	None
58	–	Infection	Deep thigh veins	No repeat imaging	–	–	None
59	–	Pain	N/A	Ultrasound	Same day	Negative	None
64	–	Infection	Common femoral vein, external iliac vein, femoral vein	No repeat imaging	–	–	Yes
66	–	Infection	Femoral vein and popliteal vein	Ultrasound	Same day	Negative	None
74	Active malignancy	Infection	Femoral vein	CT	8 days	Negative	None
77	Active malignancy	Infection	Femoral vein	Ultrasound	2 days	Negative	Yes
83	–	Infection	Femoral vein	Ultrasound	1 day	Negative	Yes
85	–	Hematoma/bleeding	Common femoral vein	Ultrasound	–	Negative	None
88	Active malignancy	Pain, swelling	Femoral vein and popliteal vein	Ultrasound	–	Positive	Yes

*N/A*, Not applicable.

**Fig:**
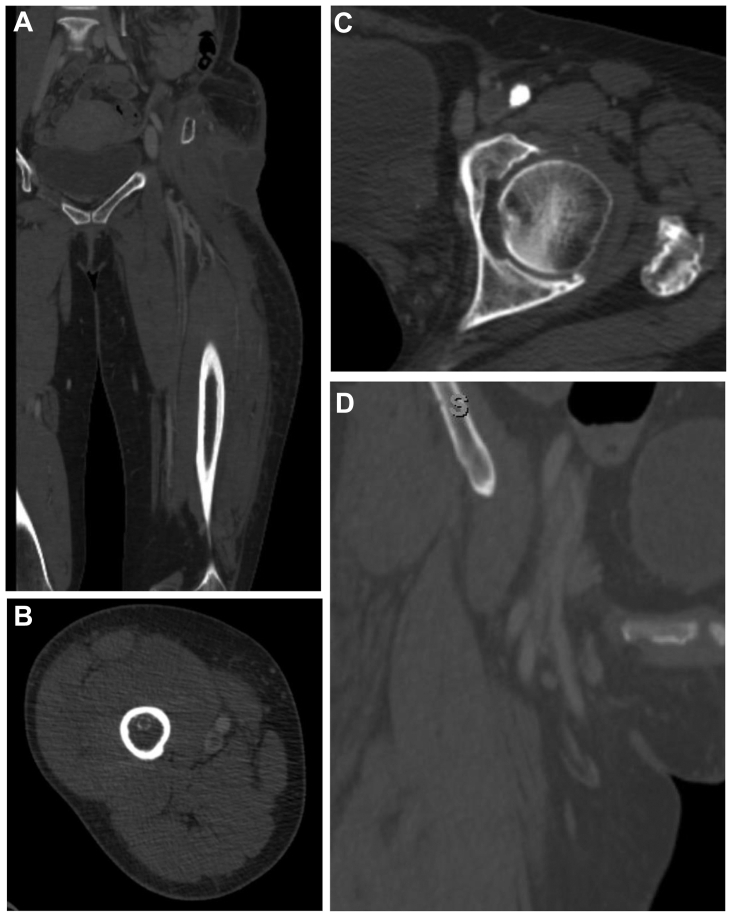
Representative images from CT scans positive for the diagnosis of DVT from four patients: **(A)** patient 77; **(B)** patient 85; **(C)** patient 23; **(D)** patient 53. *CT*, Computed tomography; *DVT*, deep venous thrombosis.

Additional imaging was ordered for 11 of the 13 patients, 9 of which were duplex studies and 2 were CT studies. The 9 duplex studies were all ordered for evaluation or confirmation of DVT in the location of the incidental CT finding. Of the 11 patients who had additional imaging, 2 (1 US and 1 CT study) were concordant with the initial CT, whereas 9 demonstrated no DVT. Of the two patients who did not receive any additional imaging, one did not have any DVT treatment initiated and the other was initiated on a 3-month course of therapeutic anticoagulation with no associated complications and no repeat imaging. Of the nine patients whose subsequent imaging did not demonstrate DVT, seven did not receive any anticoagulation. Among the two patients with discordant imaging findings, one patient was initiated on a short course of anticoagulation, but this was discontinued when the subsequent imaging did not demonstrate DVT. One patient was initiated on therapeutic heparin despite a negative venous duplex, and he developed multiple bleeding complications, including a large hematoma that required blood product transfusion as well as a gastrointestinal bleed that required discontinuation of anticoagulation and IVC filter placement. None of the patients with an incidental diagnosis of DVT, with or without anticoagulation, experienced progression to more proximal DVT or PE, stroke or transient ischemic attack, or death (Table III).

## Discussion

In this single-institution retrospective study, the 10-year incidence of DVT was 0.22% among all lower extremity CT studies. The overall published annual incidence of all lower extremity DVTs is approximately 1.6 per 1000 patients (0.16%).^13^ This incidence is significantly higher among patients with risk factors for DVT, including prior DVT, malignancy, chronic medical conditions, and hypercoagulable disorders.^14^

In particular, hospitalized patients are among the most at-risk populations for the development of VTE. Reasons for this include the proinflammatory state associated with chronic illness, vessel wall damage due to acute medical issues or surgical interventions, and stasis due to immobility. Estimates on the incidence of VTE in hospitalized patients are variable, with some reports suggesting that up to 20% of patients admitted to inpatient medicine and 40% of those admitted to surgery will develop a VTE.^3^ However, a cohort study from 2013 to 2021 found that of 1,112,014 medical (non-ICU) admissions, there were 13,843 hospital-acquired VTE (HA-VTE) events, corresponding to an incidence of only 1.2%.^15^ The study did not indicate whether these HA-VTE events occurred in only symptomatic individuals or if incidentally found DVTs were also included. The wide discrepancy in the incidence of HA-VTE can be, in part, explained by the fact that the latter study did not include ICU patients, who have a much higher risk of DVT due to critical illness and prolonged immobility.^16^ In addition, in the latter cohort study, patients who had DVT diagnosed before hospitalization or diagnosed around the time of admission to the hospital were excluded from the data, potentially providing another etiology for the discrepancy.^15^ It is unclear if these patients with a history of DVT were excluded from the incidence rates noted for the former study’s hospitalized patients with VTE.

The incidence of DVT diagnosis in the general outpatient population is not well established. The lower incidence of DVT identified in this study compared with the figures noted for inpatients above may be additionally explained by the unrestrictive inclusion criteria, incorporating all CT studies of the lower extremity performed in the inpatient, outpatient, and emergency department settings. The rate of DVT diagnoses in these latter two cohorts is likely lower, though this has not been well studied.

In this study, the rate of incidental DVTs identified on CT studies of the lower extremity was low at 0.08%. On review of the literature, the evidence surrounding the frequency of incidental CT-detected DVTs is sparse. Limited studies focus on patients with high-risk factors, such as malignancies. In the cancer population, the occurrence of an incidentally diagnosed VTE is not uncommon. In fact, some studies report that nearly half of all cancer-related VTEs are incidentally detected during routine imaging, though this is not limited to DVTs diagnosed in the extremities.^17^ In one study, Ageno et al^18^ evaluated the prevalence of incidentally diagnosed DVT in patients undergoing routine abdominal CT imaging and found that nearly 2% of the patient population had clinically silent DVTs in the lower extremities or abdominal vessels.

Although rare, CT-detected DVT may continue to be a growing issue. To evaluate the accuracy of CT as a method of evaluating proximal DVTs, Thomas et al^12^ conducted a meta-analysis of 13 articles examining patients between 1996 and 2004 who were clinically suspected of having a VTE and underwent testing for DVT using CT with US imaging or venography as a reference. Their analysis found a range of sensitivities and specificities for CT across each study, from 71% to 100% and 93% to 100%, respectively. The authors in this study acknowledge, however, that most patients within the published literature are typically imaged because of a clinical suspicion for PE. As such, this may increase the pretest probability for DVT and therefore alter the likelihood of the positive study finding.^19^ Indeed the CHEST guidelines have critiqued the heterogeneity of the studies included in this meta-analysis, citing the overall qualities of the studies as low. Because of this, the CHEST guidelines recommend against the routine use of CT venography for the diagnosis of DVT in patients with suspected first or recurrent lower extremity DVT.^20^

Furthermore, CT may be even less reliable when used for the diagnosis of DVT when DVT is not suspected. The quality of images for the diagnosis of any specific condition is dependent on study and patient factors, including contrast medium injection duration and velocity, vascular patency and structure, organ perfusion, artifact sources such as adjacent orthopedic hardware, and pharmacokinetics.^10^^,^^11^^,^^17^ As such, the accuracy of CT in diagnosing conditions such as DVT, which are highly dependent on contrast delivery and timing, may not be optimal when the primary purpose of the CT was not to evaluate for VTE.

In this study, the most common locations for incidental DVTs were the femoral (46%) and common femoral veins (31%). This differs from the reported rates of anatomic involvement for lower extremity DVTs, which were noted to be primarily distal veins (40%), followed by common femoral and femoral (both 20%), popliteal (16%), and iliac (4%) veins.^13^ There are limited studies on the anatomic distribution of incidentally found DVTs. One study by Di Nisio et al^21^ found that over half of incidental DVTs were located in distal veins. However, this study was specific to patients with cancer. The reason for the difference in DVT locations found in this study is unclear. Imaging resolution for the evaluation of small vascular structures such as calf veins can be poor, especially if there is limited contrast enhancement of these vessels. In addition, the relatively higher rates of DVT in the common femoral and femoral veins may reflect artifacts created by contrast timing as the common femoral and proximal femoral veins are particularly affected by issues such as focal reflux at the saphenofemoral junction or contrast mixing between the deep and superficial venous systems of the lower extremity. Indeed, on repeat imaging, the majority of incidentally noted DVTs in this study were ultimately not seen on repeat imaging, lending strength to this possibility.

The CHEST guidelines provide no recommendations for repeat imaging for patients who have incidentally found DVTs on CT imaging. Historically, contrast venography has been considered the gold standard for the diagnosis of DVT, with visualization of a constant intraluminal filling defect noted in more than one view considered diagnostic of DVT. However, there are risks associated with its invasive nature and the procedure can be a contraindication in patients with significant renal dysfunction, severe contrast allergies, or lower extremity wounds/injuries that limit distal vein cannulation. Finally, inadequate imaging can limit utility with up to 20% of venograms resulting in inadequate or incomplete visualization of the venous segments of interest.^20^ As a result, noninvasive modalities, including venous US imaging, have supplanted contrast venography in the diagnosis of DVT. Duplex US imaging provides excellent sensitivity (with the combined use of color Doppler to identify areas of flow deficit and continuous wave Doppler flow to identify up- and downstream flow restriction) and specificity (when compression B-mode is used to assess for areas of incompressibility) for the diagnosis of DVT, though accuracy is better for proximal DVTs as compared with isolated distal DVTs.^7^^,^^20^ Based on this evidence, the CHEST guidelines recommend D-dimer or US imaging for workup of DVT in patients with low or moderate clinical suspicion and US imaging for the diagnosis of DVT over contrast venography in patients with high clinical suspicion for DVT. CT or magnetic resonance venography is only recommended as an alternative in patients for whom US imaging is impractical.

In patients with moderate or high clinical suspicion but negative US imaging, high-sensitivity D-dimer or repeat US imaging in 1 week is recommended.^20^ However, in the cases where DVT is found on CT imaging of a patient in whom DVT was not suspected, there is no guideline-recommended next step for follow-up testing or repeat imaging. Therefore, clinicians are ultimately tasked with determining the next steps for their patients. As evidenced in this study, this results in an array of imaging pathways and varied outcomes.

For diagnosed DVTs, the CHEST guidelines recommend at least 3 months of anticoagulation for patients with proximal lower extremity DVT, regardless of symptomatology, with the choice of treatment dependent on social, economic, and clinical factors as well as the presence of cancer. The need for further extension depends on clinical factors such as the presence of a provoking event and the patient’s risk profile. In patients with a distal lower extremity DVT, the decision for repeat imaging vs treatment depends on the presence or absence of symptoms.^22^

The CHEST guidelines currently do not specifically distinguish the management of incidentally found DVTs from DVTs diagnosed in patients where there is a high index of suspicion. In this study, this resulted in a wide variety of treatment decisions with differing patient outcomes. Among the patients with incidental DVTs, five were started on anticoagulation. The two patients who had DVT confirmed on subsequent imaging completed an appropriate course of anticoagulation and did not experience any complications from their DVT or anticoagulation. One patient who did not have repeat imaging ultimately completed a 3-month course of anticoagulation and also did not experience any complications associated with their DVT or with their treatment. Among the two patients whose repeat imaging did not demonstrate DVT, one had the anticoagulation discontinued after the negative repeat study, whereas the other had continuation of the anticoagulation and experienced significant bleeding complications.

Although this risk of major hemorrhage on anticoagulation for DVT is generally outweighed by the benefits of preventing proximal extension and PE, the bleeding risk is not insignificant for patients on anticoagulation. The rates of significant bleeding complications for patients on anticoagulation for DVT vary greatly depending on the patient’s risk factors, which include age, hypertension, renal disease, liver disease, prior stroke, prior bleeding, alcohol/drug abuse, and cancer, among others.^23^^,^^24^ For patients with no risk factors for hemorrhage, the risk of major bleeding is 0.8% per year. In patients with one risk factor, that rate is doubled to 1.6% per year. In patients with two or more risk factors, the rate of major bleeding is greater than 6.5% per year.^25^ Although most patients with incidental DVTs in this study did well, one patient did have significant bleeding complications from anticoagulation, suggesting that there is a cost to these incidental diagnoses. In their recommendation against the routine use of CT venography for diagnosis of DVT, the CHEST guidelines specifically note the lack of management studies to determine the consequences of using CT venography in practice.^20^

This study provides some of these much needed management data and serves as an initial evaluation of the rare clinical finding of incidentally diagnosed DVT on contrasted CT scan of the lower extremity, which has not previously been well described. Based on the findings of this study as well as the CHEST guidelines recommendations against the use of a CT venogram for the diagnosis of lower extremity DVT, the authors of this study would recommend the use of confirmatory testing, ideally with noninvasive methods such as D-dimer or US imaging, for patients incidentally diagnosed with DVT on contrasted CT. However, additional study is needed to provide evidence to support the management recommendations for incidental diagnosis of DVT on CT imaging of the lower extremities. Specifically, given the rarity of this finding, large multicentered or database studies are likely needed to adequately power an investigation on the ideal timing of confirmatory studies, the best modality, in terms of both accuracy and costs, as well as next steps after a negative confirmatory study.

The limitations of this study include its single institution, which may limit the generalizability of these findings. Abdominal-only CT scans were not included in this study, which may result in missed iliac DVTs. However, given that DVTs in this location are less common, as noted above, this would not be expected to significantly alter the findings of this study. Furthermore, given the relative rarity of DVTs identified by CT, this study was not powered to confirm any patterns identified in the diagnosis, treatment, and outcomes of these patients. In addition, algorithmic software was used to analyze patient imaging reports to identify mention of DVT, which could be subject to error. The criteria for incidental DVT diagnosis were dependent on appropriate documentation on (1) the absence of DVT in the same location and (2) a study indication that did not identify concern for DVT or PE. However, it is possible that patients had prior DVTs that were diagnosed outside of the available medical record system or that prior DVT locations were incorrectly reported. In addition, the degree of detail may vary within the indication documented in a study order. Finally, data were collected retrospectively through a chart review. Patient care is a partnership between health care providers and patients. Therefore, the intricacies of conversations about repeat imaging and anticoagulation, which may influence decisions made by providers and their patients, may not have been completely captured on notes and orders.

## Conclusions

This study found that the incidental diagnosis of DVT by CT of the lower extremity is rare. Guidelines recommend against the routine use of a CT venogram for the diagnosis of suspected DVT, but currently no guidelines exist to guide additional workup or management after an incidental DVT identified on CT imaging. As a result, clinicians pursue a variety of different diagnostic and treatment pathways, some of which may lead to detrimental effects on patient outcomes. Repeat imaging evaluation may have some value in these cases, though additional study is needed to better define the best confirmatory study, in terms of both accuracy and costs. Given the rarity of this finding, large, multicenter studies may be needed to clarify a clinical standard after a diagnosis of incidental DVT on CT studies of the lower extremity.

## Author Contributions

Conception and design: PB, DC, RG, MK

Analysis and interpretation: PB, DC, MK

Data collection: PB, DC, MK

Writing the article: PB, DC, MK

Critical revision of the article: PB, DC, RG, MK

Final approval of the article: PB, DC, RG, MK

Statistical analysis: Not applicable

Obtained funding: Not applicable

Overall responsibility: MK

PB and DC share co-first authorship.

## Disclosures

None.
